# Heterogeneous IL-9 Production by Circulating Skin-Tropic and Extracutaneous Memory T Cells in Atopic Dermatitis Patients

**DOI:** 10.3390/ijms25168569

**Published:** 2024-08-06

**Authors:** Irene García-Jiménez, Lídia Sans-de San Nicolás, Laia Curto-Barredo, Marta Bertolín-Colilla, Eloi Sensada-López, Ignasi Figueras-Nart, Montserrat Bonfill-Ortí, Antonio Guilabert-Vidal, Anna Ryzhkova, Marta Ferran, Giovanni Damiani, Tali Czarnowicki, Ramon M. Pujol, Luis F. Santamaria-Babí

**Affiliations:** 1 Immunologia Translacional, Departament de Biologia Cellular, Fisiologia i Immunologia, Facultat de Biologia, Universitat de Barcelona (UB), Parc Científic de Barcelona (PCB), 08028 Barcelona, Spain; irenegarciajimenez@ub.edu (I.G.-J.);; 2 Departament de Dermatologia, Hospital del Mar, Institut Hospital del Mar d’Investigacions Mèdiques (IMIM), Universitat Autònoma de Barcelona (UAB), 08003 Barcelona, Spain; 3 Departament de Dermatologia, Hospital de Bellvitge, Universitat de Barcelona (UB), 08907 L’Hospitalet de Llobregat, Spain; 4 Departament de Dermatologia, Hospital General de Granollers, 08402 Granollers, Spain; 5 Italian Center of Precision Medicine and Chronic Inflammation Milan, 20122 Milan, Italy; 6 Department of Biomedical, Surgical and Dental Sciences, Faculty of Medicine and Surgery, University of Milan, 20122 Milan, Italy; 7 Dr. Phillip Frost Department of Dermatology and Cutaneous Surgery, Miller School of Medicine, University of Miami, Miami, FL 33136, USA

**Keywords:** atopic dermatitis, cutaneous lymphocyte-associated antigen, extracutaneous, heterogenous, house dust mite, interleukin-9, skin-tropic, staphylococcal enterotoxin B

## Abstract

Interleukin (IL)-9 is present in atopic dermatitis (AD) lesions and is considered to be mainly produced by skin-homing T cells expressing the cutaneous lymphocyte-associated antigen (CLA). However, its induction by AD-associated triggers remains unexplored. Circulating skin-tropic CLA^+^ and extracutaneous/systemic CLA^−^ memory T cells cocultured with autologous lesional epidermal cells from AD patients were activated with house dust mite (HDM) and staphylococcal enterotoxin B (SEB). Levels of AD-related mediators in response to both stimuli were measured in supernatants, and the cytokine response was associated with different clinical characteristics. Both HDM and SEB triggered heterogeneous IL-9 production by CLA^+^ and CLA^−^ T cells in a clinically homogenous group of AD patients, which enabled patient stratification into IL-9 producers and non-producers, with the former group exhibiting heightened HDM-specific and total IgE levels. Upon allergen exposure, IL-9 production depended on the contribution of epidermal cells and class II-mediated presentation; it was the greatest cytokine produced and correlated with HDM-specific IgE levels, whereas SEB mildly induced its release. This study demonstrates that both skin-tropic and extracutaneous memory T cells produce IL-9 and suggests that the degree of allergen sensitization reflects the varied IL-9 responses in vitro, which may allow for patient stratification in a clinically homogenous population.

## 1. Introduction

Interleukin (IL)-9 is involved in Th2-mediated allergic inflammation [1], and it plays a pleiotropic role in allergic airway diseases, promoting eosinophil and mast cell infiltration, IgE secretion, and mucus production [2,3].

T lymphocytes, type 2 innate immune cells (ILC2s), mast cells, neutrophils and eosinophils produce IL-9, but the major source of IL-9 production seems to be Th cells [1,4]. IL-9 binds to the IL-9 receptor (IL-9R), a heterodimer formed by the common γc receptor and the cytokine-specific IL-9 receptor α-chain (IL-9Rα), for signal transduction through the JAK/STAT, PI3K, and MAPK pathways [4]. Multiple cell types express IL-9R, including hematopoietic cells [1,4], keratinocytes [5], and melanocytes [6].

In humans, IL-9-producing CD4^+^ T cells have been described as a subpopulation of peroxisome proliferator-activated receptor-gamma (PPAR-γ)^+^ Th2 cells expressing the chemokine receptors CCR4 and CCR8 [7]. They exhibit transient IL-9 production, heightened IL-13, and IL-5 secretion [7], a tendency to express the skin-homing receptor known as cutaneous lymphocyte-associated antigen (CLA) [8,9], and are typically located in skin lesions of atopic dermatitis (AD), psoriatic, and contact dermatitis patients [7,9]. Interestingly, blood phenotyping studies reported increased Th9 frequencies in peripheral blood of distinct allergic airway conditions [10,11] and CLA^+^ Th9 populations in several inflammatory skin diseases [6,12,13,14].

IL-9 is increased in the blood/sera and skin of AD patients and is associated with disease severity, total IgE, and CCL17 levels in blood [15,16,17,18]. Moreover, IL-9R expression is enhanced in lesional and non-lesional AD skin [5,15,19] and is related to disease severity [20]. Additionally, Th9 differentiation factors Itk and PU.1 are increased in AD lesional skin and peripheral blood mononuclear cells (PBMCs), respectively [17,18,21,22,23]. Descriptive studies on PBMCs have shown IL-9 production by CD4^+^/CD8^+^ within skin-homing/CLA^+^ and systemic/CLA^−^ compartments in AD patients [24,25,26].

Thus far, in humans, IL-9 production by T cells has been mainly studied via polyclonally activated PBMCs and intracellular flow cytometry analysis [7,8,12,13,14,24,25,26]. However, whether IL-9 can be induced by pathological triggers associated with AD, such as house dust mite (HDM) allergens or staphylococcal enterotoxin B (SEB), specifically in CLA^+^/CLA^−^ memory T cells, remains unexplored. Additionally, the relationship between this IL-9 production and the clinical status of the patients has not been characterized.

Our results expand the knowledge of the role of IL-9 in AD showing that it may be produced by CLA^+^ and CLA^−^ memory T cells stimulated by either HDM or SEB. Furthermore, it has been observed that HDM triggers substantial and varied IL-9 production in vitro, and this production closely resembles the level of allergen sensitization observed in AD patients in a clinical context.

## 2. Results

### 2.1. In AD Patients, HDM-Induced CLA^+^/CLA^−^ T-Cell IL-9 Levels Are Higher Than Th2, Th1, and Th17-Related Cytokines and Correlate with Plasma IgE

**Figure 1:**
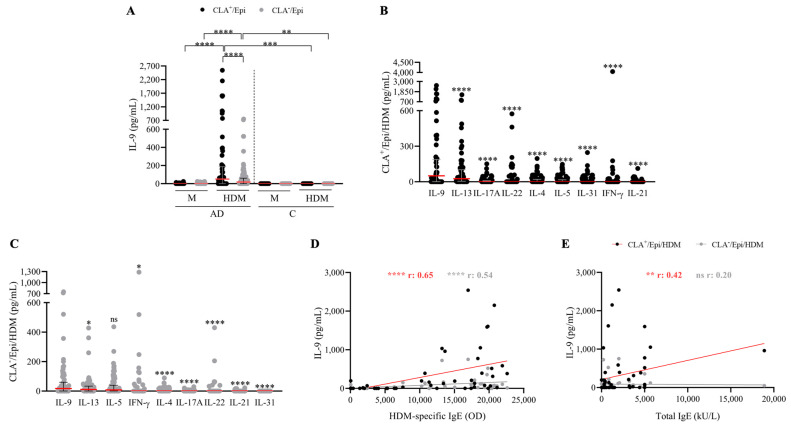
**HDM induces IL-9 production by both skin-tropic CLA^+^ and non-tropic CLA^−^ memory T cells at higher level than other proinflammatory cytokines, which correlates with HDM-specific IgE plasma levels.** (**A**) IL-9 (pg/mL) produced in basal conditions (M) or stimulated with HDM in AD- (n = 52) and C-derived (n = 12) CLA^+^/Epi and CLA^−^/Epi cocultures after 5 days. IL-4, IL-5, IL-9, IL-13, IL-17A, IL-31, IFN-γ (n = 52), IL-21 (n = 46), and IL-22 (n = 45) cytokines were simultaneously quantified in (**B**) CLA^+^/Epi and (**C**) CLA^−^/Epi AD cocultures. Cytokines were sorted according to the median production and compared in relation to IL-9 production levels. Red line indicates the median. Correlations between IL-9 (pg/mL) and (**D**) HDM-specific, and (**E**) total IgE plasma levels (n = 51). AD, atopic dermatitis; C, control subjects; CLA, cutaneous lymphocyte-associated antigen; Epi, epidermal cells; HDM, house dust mite; M, untreated; OD, optical density. ns: *p* > 0.05; * *p* < 0.05; ** *p* < 0.01; *** *p* < 0.001; **** *p* < 0.0001.

HDM-specific induction of IL-9 production was assessed in circulating CLA^+^ or CLA^−^ memory T cells cocultured with autologous lesional epidermal cells from AD (n = 52) and control (n = 12) individuals. IL-9 secretion was preferentially observed in CLA^+^ compared to CLA^−^ T-cell cocultures in AD and was not detected in control-derived CLA^+/−^ T cell cocultures stimulated with HDM (Figure 1A). IL-9 levels were measured along with other AD-associated mediators (IL-4, IL-5, IL-13, IL-17A, IL-21, IL-22, IL-31, and IFN-γ). Upon HDM activation, IL-9 was found to be the most produced cytokine within the CLA^+^ T-cell population and was produced at equal levels to IL-5 in the CLA^−^ fraction (Figure 1B,C).

A positive association between HDM-specific IgE and IL-9 production by HDM-induced CLA^+^ (r = 0.65, *p* < 0.0001) and CLA^−^ (r = 0.54, *p* < 0.0001) T-cell cocultures (Figure 1D) was found, whereas a correlation with total IgE was only detected in the skin-homing compartment (r = 0.42, *p* = 0.002; Figure 1E). Interestingly, HDM-specific IgE levels were also directly associated with the production levels of the Th2 cytokines quantified in the cocultures (Figure S1). No further correlations were observed between IL-9 and other clinical features (Table S1).

### 2.2. HDM-Induced IL-9 Secretion by Memory T Cells Requires the Contribution of Autologous Epidermal Cells and Depends on HLA-Mediated Presentation

**Figure 2:**
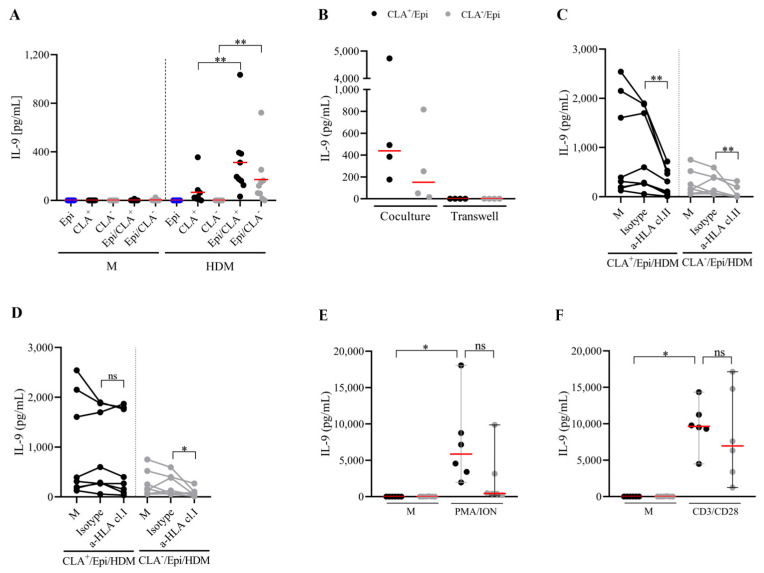
**HDM-activated memory T cells require cell-to-cell contact with epidermal cells for IL-9 production, which primarily depends on HLA class II molecules.** (**A**) Epidermal cells, CLA^+/−^ T cells, and CLA^+/−^/Epi cocultures were left untreated or stimulated with HDM, and IL-9 (pg/mL) was measured at day 5. (**B**) HDM-stimulated (n = 4) CLA^+/−^ T cells and epidermal cells were added in the upper and lower chamber of the transwell plate (right) or cocultured together (left), and IL-9 (pg/mL) was quantified on day 5. (**C**) HLA class I and (**D**) class II molecules were neutralized in HDM-activated CLA^+/−^/Epi cocultures on day 0, and IL-9 levels (pg/mL) were compared to isotype values on day 5 (n = 8). Cytokine content under HDM stimulation without neutralizing antibodies (M) are shown. CLA^+/−^/Epi cocultures were polyclonally activated with (**E**) PMA/ION and (**F**) anti-CD3/CD28 beads, and IL-9 (pg/mL) was measured on day 5 (n = 6). Red line indicates the median. Cl, class; CLA, cutaneous lymphocyte-associated antigen; Epi, epidermal cells; HDM, house dust mite; HLA, human leukocyte antigens; ION, ionomycin; M, untreated; PMA, phorbol myristate acetate. ns: *p* > 0.05; * *p* < 0.05; ** *p* < 0.01.

To understand the participation of autologous lesional epidermal cells in IL-9 production in the coculture, T cells and epidermal cells were cultured alone or together for 5 days. Epidermal cells enhanced HDM-induced IL-9 production by memory T cells (Figure 2A), which seems to be produced in a time-dependent manner (Figure S2). Moreover, transwell assay further revealed that direct cell-to-cell contact between epidermal cells and T cells was necessary for cytokine production (Figure 2B). Additionally, HLA class I and class II were neutralized in the cocultures. Blocking of MHC class II reduced around 70 and 80% IL-9 production in CLA^+^ and CLA^−^ cocultures, respectively. MHC class I blockade also affected cytokine release by about 50% in the CLA^−^ fraction, but it failed to inhibit the production of IL-9 by the CLA^+^ population (25% of inhibition) (Figure 2C,D). No differences in IL-9 content between polyclonally activated CLA^+^/Epi and CLA^−^/Epi cocultures were found (Figure 2E,F).

### 2.3. AD Patients Producing HDM-Induced IL-9 in CLA^+^/CLA^−^ Memory T Cells Present Higher Levels of HDM-Specific and Total IgE

**Figure 3:**
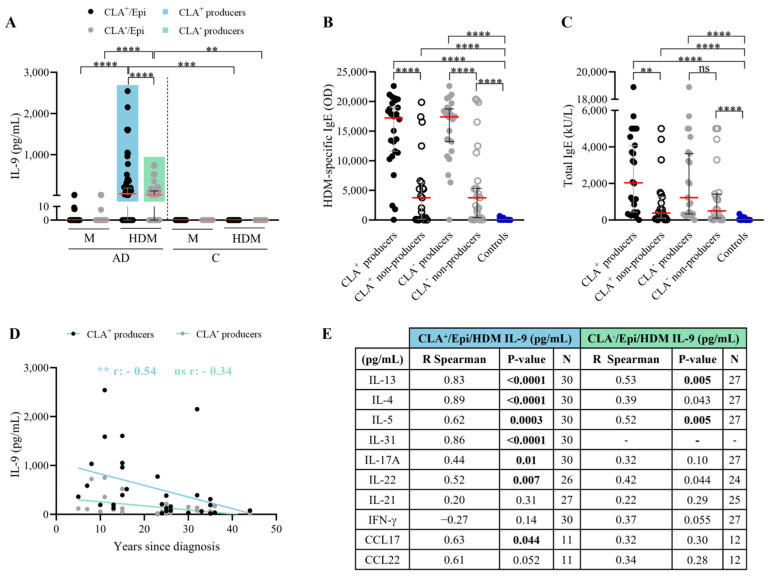
**Patients with HDM-induced CLA^+^ T cell IL-9 response have elevated specific and total IgE plasma levels; their IL-9 levels decrease over the course of the disease and, within the CLA^+^ subset, IL-9 correlates with cytokines of Th2- and Th17/22-induced inflammation.** (**A**) AD patients were stratified according to IL-9 production by HDM-stimulated CLA^+^ T (producers n = 30, non-producers n = 22) and CLA^−^ T cells (producers n = 27, non-producers n = 25). (**B**) Specific IgE (OD) and (**C**) total IgE (kU/L) plasma levels were compared between IL-9 producers and non-producers in the CLA^+^ (n = 28 and 26, respectively) and CLA^−^ (n = 21 and 23, respectively) subsets in AD patients, and control volunteers (n = 25). Red lines indicate the median. Correlation between IL-9 (pg/mL) produced by HDM-activated CLA^+/−^ T cell cocultures and (**D**) years since diagnosis and (**E**) IL-13, IL-4, IL-5, IL-31, IL-17A, IL-22, IL-21, IFN-γ, CCL17, and CCL22 (pg/mL) within CLA^+^ (n = 11–30) and CLA^−^ (n = 12–27) IL-9 producers. Bold values indicate significant data. AD, atopic dermatitis; C, control subjects; CLA, cutaneous lymphocyte-associated antigen; Epi, epidermal cells; HDM, house dust mite; M, untreated; OD, optical density. ns: *p* > 0.05; ** *p* < 0.01; *** *p* < 0.001; **** *p* < 0.0001.

IL-9 was detected in 58% and 52% of HDM-stimulated CLA^+^ and CLA^−^ cocultures, respectively (Figure 3A). AD patients were segregated into IL-9 producers and non-producers. IL-9-producing (either CLA^+^/CLA^−^) AD patients exhibited the highest specific and total IgE levels (Figure 3B,C). Although non-producers had lower IgE levels, their degree of allergen sensitization and allergic status was higher than controls. Moreover, IL-9 response to HDM tended to correlate with HDM-specific IgE levels in IL-9 producers, specifically among the CLA^+^ subset (Table S4). Additionally, HDM-induced CLA^+^ T cell IL-9 production indirectly correlated with AD duration (r = −0.54, *p* = 0.002) (Figure 3D). No further associations were found between IL-9 and AD clinical characteristics (Table S2).

Within the IL-9 producers, the IL-9 content from the CLA^+^ T cell cocultures correlated with IL-13 (r = 0.83, *p* < 0.0001), IL-4 (r = 0.89, *p* < 0.0001), IL-31 (r = 0.86, *p* < 0.0001), IL-5 (r = 0.62, *p* = 0.0003), IL-17A (r = 0.44, *p* = 0.01), IL-22 (r = 0.83, *p* = 0.007), and CCL17 (r = 0.63, *p* = 0.04), and closely associated with CCL22 (r = 0.61, *p* = 0.05), whereas in the CLA^−^ T cell cocultures, IL-9 content was strictly associated with IL-13 (r = 0.53, *p* = 0.005) and IL-5 (r = 0.52, *p* = 0.005) (Figure 3E). Likewise, IL-9 producers showed higher levels of IL-4, IL-5, IL-13, IL-17A, IL-21, IL-22, IFN-γ, IL-31, and IL-21 production, the latter two significant only in the CLA^+^ subset, compared to non-producers (Tables S3 and S4). Interestingly, CLA^+^ IL-9-producing patients presented elevated LDH serum levels (Tables S3 and S4).

### 2.4. SEB Induces Low IL-9 Production in CLA^+^ and CLA^−^ Memory T Cells Compared to Other Proinflammatory Cytokines

**Figure 4:**
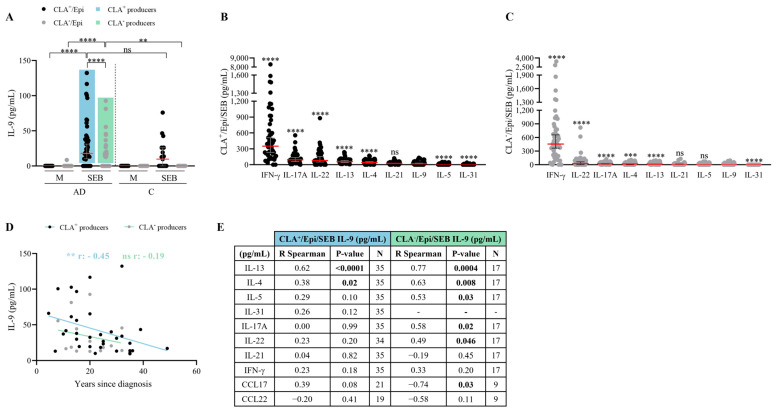
**SEB triggers mild IL-9 production in both CLA^+^ and CLA^−^ memory T cells.** (**A**) IL-9 (pg/mL) produced in basal conditions (M) or stimulated with SEB in AD- (n = 50) and C-derived (n = 17) CLA^+^/Epi and CLA^−^/Epi cocultures after 24 h. AD patients were stratified according to SEB-induced IL-9 response. Blue and green boxes show those AD patients producing IL-9 by CLA^+^ (n = 35) and CLA^−^ (n = 17) T cells, respectively. (**B**) In CLA^+^/Epi and (**C**) CLA^−^/Epi AD cocultures IL-4, IL-5, IL-9, IL-13, IL-17A, IL-31, IFN-γ (n = 52), IL-21 (n = 46), and IL-22 (n = 45) levels were simultaneously quantified. Cytokines were sorted according to the median production and compared to IL-9 production levels. Red lines indicate the median. Correlations of IL-9 levels (pg/mL) in HDM-activated CLA^+^ and CLA^−^ T cell cocultures and (**D**) years since diagnosis (n = 33 for CLA^+^, n = 16 for CLA^−^), (**E**) IL-13, IL-4, IL-5, IL-31, IL-17A, IL-22, IL-21, IFN-γ, CCL17, and CCL22 (pg/mL) within patients with IL-9 production. Bold values indicate significant data. AD, atopic dermatitis; C, control subjects; CLA, cutaneous lymphocyte-associated antigen; Epi, epidermal cells; M, untreated; SEB, staphylococcal enterotoxin B. ns: *p* > 0.05; ** *p* < 0.01; *** *p* < 0.001; **** *p* < 0.0001.

In both AD and control subjects, SEB induced preferential IL-9 secretion in CLA^+^ compared to CLA^−^ memory T cells; however, IL-9 induction was more prominent in AD patients, particularly in the CLA^−^ compartment (Figure 4A). Compared to the effector cytokines IL-13, IL-4, IL-17A, IL-22, and IFN-γ, SEB activation led to lower levels of IL-9 induction (Figure 4B,C). Surprisingly, despite the disparate IL-9 secretion triggered by HDM- and SEB-activated memory T cells, a positive correlation was found between HDM- and SEB-induced IL-9 responses by circulating memory CLA^+^ and CLA^−^ T cells (Figure S3). No correlations were found between IL-9 and *S. aureus*- and SEB-IgE plasma levels nor other AD clinical characteristics (Table S5).

SEB induced CLA^+^ and CLA^−^ T cell-mediated IL-9 production in 70% and 34% of AD patients, respectively, as indicated by the blue box for the former and the green box for the latter (Figure 4A). A strong negative correlation was observed between disease duration and CLA^+^ T-cell IL-9 secretion in the IL-9 producers (r = −0.45, *p* = 0.009; Figure 4D). No additional clinical correlations were identified (Table S6). In the IL-9 producers, the IL-9 levels derived from the skin-homing cocultures was exclusively linked to the Th2 mediators IL-13 (r = 0.62, *p* < 0.0001) and IL-4 (r = 0.38, *p* = 0.02), and closely linked to CCL17 (r = 0.39, *p* = 0.08), whereas the systemic CLA^−^/Epi IL-9 response was related to IL-13 (r = 0.77, *p* = 0.0004), IL-4 (r = 0.63, *p* = 0.008), IL-5 (r = 0.53, *p* = 0.03), IL-17A (r = 0.58, *p* = 0.02), and IL-22 (r = 0.49, *p* = 0.05);interestingly, it negatively associated with CCL17 (r = −0.74, *p* = 0.03) (Figure 4E). Additionally, IL-9-producing AD patients showed increased production of all cytokines assessed, except for IL-31 in both CLA^+^ and CLA^−^ subsets and IFN-γ production increased only in the former subset (Tables S7 and S8).

## 3. Discussion

Currently, it is believed that the production of IL-9 by memory T cells is transiently and primarily generated by skin-tropic effector memory Th9 lymphocytes [8]. Most reports addressing this subset rely on naïve T cells differentiated in vitro or memory T cells polyclonally activated [7]. In AD, IL-9 secretion has been analyzed intracellularly in CD4^+^/CD8^+^ CLA^+^ and CLA^−^ memory T cells with no differences in Th9/Tc9 frequencies between AD and control individuals [24,25,26]. Czarnowicki et al. showed that normal and AD progression demonstrate an age-related increase in IL-9 levels, followed by a decline in adulthood. Additionally, IL-9^+^ -cell subsets displayed the strongest association to AD disease measures [26]. To additionally explore the possible role of IL-9 in AD, we have used an ex vivo coculture system made of patient’s purified circulating memory T cells and autologous lesional epidermal cells that has previously enabled the association of T-cell functions with clinical features in AD [27,28] and psoriasis [29]. Control subjects recruited in this study were non-age- and sex-matched with AD patients, which remains an epidemiological limitation of the study.

CLA^+^ T cells represent a memory T-cell subset involved in the cutaneous immune response and constitutes a relevant cell population in AD pathogenesis [30]. Circulating CD3^+^ CD4^+^ CD45RO^+^ CLA^+^ T cells abundantly infiltrate AD skin [31] and increased IL-9 expression and Th9 frequencies are found in AD lesions [8,15]. However, IL-9-producing T cells have been related not only to skin diseases, but also to extracutaneous allergic conditions [1]. In fact, in asthmatic patients the stimulation of memory T cells preferentially expands the CLA^−^ fraction [32].

Until now, memory T-cell IL-9 response in human AD has been poorly characterized and it remains unexplored whether pathological triggers associated with AD can induce the production of this cytokine. In the present study, we demonstrated that both HDM and SEB, clinically considered AD triggers, induce IL-9 production not only in skin-homing CLA^+^ memory T cells, but also in the extracutaneous CLA^−^ compartment. Additionally, upon HDM allergen exposure, we evidenced that AD patients produced more IL-9 compared to control individuals by both CLA^+^ and CLA^−^ T cells and observed cytokine production in a time-dependent manner, in contrast to previous studies performed by polyclonal activation and intracellular staining [24,25,26]. The reason for these differences may be the use of a more physiological method and the activation of HDM and SEB antigen-specific T cells. The possible clinical relevance of these results is supported by the significant correlation observed between specific IgE plasma levels and IL-9.

Allergens, such as HDM, are recognized, internalized, and presented by antigen-presenting cells (APC). In our coculture, epidermal cells serve as a plausible source of APCs, given that IL-9 production is heightened in their presence and require direct cell-to-cell contact with memory T cells for cytokine production. Moreover, the secretion of IL-9 was substantially reduced by the neutralization of MHC class II molecules. This result is in line with previous observations on allergen-specific T cells [33] and supports the concept that CD4^+^ T cells are the main cellular source of IL-9 [1]. However, within the CLA^−^ subset cytokine production was partially inhibited by the blockade of class I molecules. This observation indicates a role for CD8^+^ T cells in extracutaneous allergic inflammation, as suggested by some authors in allergen-mediated models of asthma [34,35] and deserves further exploration.

Although a recent study illustrates that *S. aureus*, including its derived toxins TSST1 and SEA, influence Th9 polarization in polyclonally activated PBMCs from control subjects [36], reports in AD are rather limited and *S. aureus*-dependent IL-9 production has not been observed [8,37]. Notably, the substantial IL-9 production observed in SEB-induced cocultures of CLA^+^ T cells, as compared to CLA^−^ T cells, can be attributed to the fact that SEB toxin directly interacts with HLA II molecules found on keratinocytes or APCs and activates CLA^+^ memory T cells expressing a particular TCR Vβ-chain [38,39,40]. However, the activation of CLA^−^ T cells by SEB has been poorly characterized and mainly limited to flow cytometry analysis [39,40,41,42]. Future efforts to explore SEB-related TCR Vβ within extracutaneous lymphocytes would be of interest for understanding the role of SEB in other diseases.

The relative levels of IL-9 and other AD-related cytokines assessed was notoriously different depending on the stimuli. In the HDM-activated T-cell cocultures, IL-9 was the most abundant cytokine produced, closely followed by IL-13, the core Th2 cytokine in AD pathogenesis [43]. Of note, the equal IL-9 and IL-5 production in the CLA^−^ T-cell coculture is of particular interest since anti-IL-5 therapy has proven to be successful in extracutaneous allergic diseases such as asthma [44], but not in AD [45], considered a CLA^+^-driven disease [30]. Conversely, SEB is a staphylococcal toxin that triggers the massive production of pro-inflammatory cytokines, such as IL-2, TNF-α and IFN-γ [46]. In line, low amounts of IL-9 were detected in SEB-activated CLA^+^/^−^ T-cell cocultures compared to IFN-γ. Although the IL-9 content released by HDM- and SEB-activated memory T cells was particularly distinct, a positive correlation was found between HDM- and SEB-induced IL-9 responses by circulating memory CLA^+^/CLA^−^ T cells. Considering that *S. aureus* sensitization is frequent in HDM-sensitized AD patients [47] and SEB facilitates allergen-specific Th2 responses [48], assessing the possible synergic effect of HDM and SEB to trigger IL-9 response as well as exploring whether other allergens and *S. aureus* toxins could induce a memory T-cell response in our model remains a limitation in this study.

In our study, the heterogeneous production of IL-9 by CLA^+^ and CLA^−^ memory T cells has enabled the definition of IL-9 producers and non-producers subjects, depending on the stimuli, and has expanded the knowledge of previous reports assessing IL-9 production in those T-cell subsets [12,13,14,24,25,26]. Slightly over half of our AD cohort produced IL-9 upon HDM exposure. Cocultures from AD patients with IL-9 production also showed increased levels of Th2, Th1, and Th17/22 cytokines in response to HDM, except for IL-31 and IL-21 in the CLA^−^ compartment since the former is CLA^+^-specific [28] and the latter is produced more by CLA^+^ T cells [49]. Two independent proteomic studies showed that the “Th1/Th2/Th17-dominant” cluster presented the highest IL-9 serum levels, supporting the direct correlations observed between skin-tropic IL-9 and IL-13, IL-4, IL-5, IL-31, IL-17A, IL-22, and CCL17 [50,51]. Conversely, IL-9 in the CLA^−^ subset was only associated with IL-13 and IL-5, highlighting the type 2 inflammation that characterizes allergic extracutaneous diseases [52]. Similarly, previous studies indicate that CD4^+^ T cells coproducing heightened IL-9, IL13, and IL-5 levels are key players in allergic airway inflammation in mice after allergen exposure [53], and gene coexpression analysis suggests that these three cytokines are coexpressed in HDM-reactive Th2 cells from HDM-allergic individuals [54]. Moreover, in AD patients who are IL-9 producers, there is an elevation in both allergen-specific and total IgE levels. This observation indicates that the extent of HDM sensitization and the overall allergic condition of the patient provide clinical support for the diverse IL-9 production seen in AD.

Upon SEB activation, 70% of AD patients from our cohort produced IL-9 by CLA^+^ T cells. *S. aureus*-colonized patients display greater serum levels of Th2 biomarkers than non-colonized patients [55], supporting the association between IL-9 and IL-4 and IL-13 in the producers group. Although most AD subjects challenged with SEB manifested CLA^+^ T-cell IL-9 response, this cytokine was mildly produced compared to the high content detected in the HDM coculture, suggesting that HDM might be a more powerful IL-9 inductor than SEB. Consistent with this notion, IL-9 together with IL-13, the second most produced cytokine upon HDM activation, showed the strongest association with HDM-specific IgE plasma levels. Nonetheless, the exact mechanisms behind the increased production of IL-9 triggered by HDM deserves further investigation.

Prior studies showed that IL-9 increases during infancy and decreases in adulthood [26] and is more abundant in pediatric-onset AD patients [56]. In addition to the current knowledge, we found that regardless of the stimuli, there was an indirect correlation between skin-tropic IL-9 production and AD duration (years since disease diagnosis), altogether suggesting that IL-9 might be a relevant mediator in the initial stages of AD development and progression.

Recent research demonstrated that IL-9 enhances aerobic glycolysis in IL-9-producing Th cells, with lactate dehydrogenase A (LDHA) being one of the most upregulated genes [57]. Interestingly, our results showed that AD patients with HDM-induced CLA^+^ T-cell-mediated IL-9 response to HDM, but not SEB, exhibited increased LDH serum levels, which opens a new line of study between the possible implication of CLA^+^ T-cell IL-9 response and glycolytic metabolism.

Patients with IL-9 production by both cutaneous and extracutaneous memory T cells in response to HDM exhibited greater HDM-specific IgE plasma levels than IL-9 non-producer AD patients and controls. Indeed, HDM-allergic AD patients displayed higher serum IL-9 than patients sensitized to other allergens [58]. Additionally, the epicutaneous application of HDM to non-lesional skin in human AD and HDM-sensitized atopic dogs induced robust IL-9 mRNA expression [59,60]. Furthermore, a single-cell transcriptomic analysis revealed that HDM-specific memory Th cells expressed greater amounts of IL-9 transcript than HDM-non-reactive memory Th cells [54].

Altogether, our findings expand the understanding of IL-9 production in AD. They highlight how pathogenic stimuli induce diverse IL-9 production patterns, involving both cutaneous and extracutaneous memory T cells, in a persistent manner, with a preference observed in patients rather than control subjects. Interestingly, “IL-9^high^” patients endotype have been recently suggested [61,62]. For this reason, we believe that these findings pave the way for a deeper understanding of the variability in IL-9 production among AD patients and underscore its potential as a candidate for targeted therapeutic interventions.

## 4. Materials and Methods

### 4.1. Patients and Biological Samples

**Figure 5:**
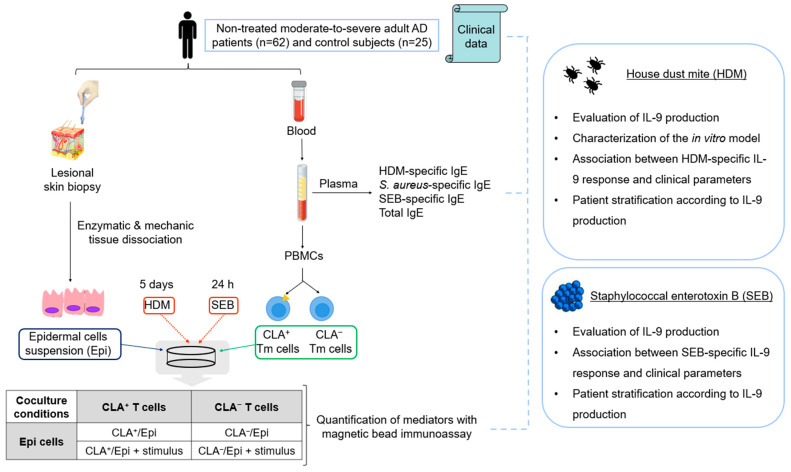
**Workflow of the study design.** AD, atopic dermatitis; CLA, cutaneous lymphocyte-associated antigen; Epi, epidermal cells; HDM, house dust mite; PBMCs, peripheral blood mononuclear cells; SEB, staphylococcal enterotoxin B; Tm, memory T cells. This figure was created using images under a Creative Commons license.

This study included 62 non-treated moderate-to-severe adult AD patients and 25 non-age- and sex-matched control individuals. Written informed consents were obtained, and human sample collection was carried out according to the institutional review board-approved protocols at the Hospital del Mar and Hospital de Granollers (Spain); and clinical information was recorded (Tables S9 and S10). Blood samples and two skin biopsies from lesional areas were obtained without any topical or systemic anti-inflammatory treatments administered for a minimum of 2–4 weeks prior to the study, respectively. The workflow of the study design can be found in Figure 5.

### 4.2. Isolation of CLA^+/−^ Memory T Cells and Epidermal Cell Suspension

CLA^+^ and CLA^−^ memory CD45RA^−^ T lymphocytes were purified from whole blood after PBMC isolation by Ficoll (GE Healthcare, Princeton, NJ, USA) gradient and three consecutive immunomagnetic separations (Miltenyi Biotech, Bergisch Gladbach, Germany) as previously described [63]. Lesional skin biopsies were incubated overnight in dispase (Corning, NY, USA) at 4 °C. The epidermal sheet was peeled off from the dermis, cut into pieces, and incubated in trypsin (Biological Industries, Kibbutz Beit Haemek, Israel) for 15 min at 37 °C. Epidermal tissue was mechanically disaggregated by gently pipetting, and the epidermal cell suspension (Epi) was transferred to fresh media (RPMI, 10% FBS, 1% penicillin-streptomycin (Sigma-Aldrich, St. Louis, MO, USA)).

### 4.3. Coculture of CLA^+/−^ Memory T Cells with Epidermal Cells and Stimulation

Coculture of 5 × 10^4^ CLA^+/−^ T cells and 3 × 10^4^ autologous epidermal cells (CLA^+^/Epi or CLA^−^/Epi, respectively) in 96-well U-bottom plates (Falcon, Corning, NY, USA) were left untreated (M) or activated at 24 h with SEB (2 μg/mL) or at 5 days with HDM extract (10 μg/mL) (kindly provided by LETI Pharma, Barcelona, Spain), and indicated with PMA (25 ng/mL) plus Ionomycin (2 μg/mL) (Sigma-Aldrich) or anti-CD3/anti-CD28 beads (1:1 bead-to-cell ratio) (ThermoFisher Scientific, Waltham, MA, USA). For non-contact coculture, epidermal cells were seeded at the bottom of a transwell 96-well plate (Corning), and T cells were placed in the cell culture insert using the mentioned amounts for each cell type, and HDM activation was performed in the same way. For neutralization assays, azide-free HLA-A/B/C (class I), HLA-DR (class II), and respective isotype IgG2a control antibodies (Biolegend, San Diego, CA, USA) were added at 10 μg/mL final concentration before HDM activation. Supernatants were collected and kept at −20 °C for cytokine quantification. For the time course experiment, supernatants were collected at different timepoints (day 1, 2, 4, and 5).

### 4.4. Cytokine Quantification

Eleven markers (IL-4, IL-5, IL-9, IL-13, IL-17A, IL-21, IL-22, IL-31, IFN-γ, CCL17, and CCL22) were measured in supernatants using the ProcartaPlex Multiplex immunoassay (Invitrogen, Waltham, MA, USA), detected using a MAGPIX plate reader (Luminex Technologies Inc., Austin, TX, USA) and analyzed with ProcartaPlex Analyst software version 1.0 (Invitrogen) using a five-parameter logistic curve. Values below the lower limit of quantification (LLOQ) were treated as zero.

### 4.5. Quantification of Total and Specific IgE against HDM, S. aureus, and SEB

HDM-specific IgE (response (OD)) and total IgE (kU/L) plasma levels were measured by ImmunoCAP (ThermoFisher Scientific).

*S. aureus*- and SEB-specific IgE (OD) plasma levels were assessed by ELISA, as previously described [27] with slight modifications. The plasma used to quantify *S. aureus* and SEB-IgE titers was previously depleted of IgG by immunodepletion. Plasma samples were diluted in 1% BSA (Biowest, Nuaillé, France) (1:5), and protein G (Sigma) was added at 150 µg/mL final concentration. After 45 min of incubation at RT on an orbital shaker, the IgG-depleted plasma was recovered by centrifugation and further diluted 50- and 2-fold in PBS-1% skimmed milk for IgE-*S. aureus* and IgE-SEB quantification, respectively. Next, alkaline phosphatase-labeled goat anti-human IgE (Sigma) was diluted 1:250 and 1:500 in PBS-1% skimmed milk for the detection of IgE against *S. aureus* and SEB, respectively. *S. aureus*- and SEB-IgE titers below the limit of detection (LOD) were treated as zero.

### 4.6. Statistical Analysis

Statistical analysis and graphical representation were conducted with GraphPad Prism software version 8.0.1 (GraphPad Software Corporation, San Diego, CA, USA). Data are generally presented as scatter dot plot and the median ± 95% confidence interval (CI). Differences between the two groups or within the same group were detected by the Mann–Whitney test and the Wilcoxon test, respectively. Spearman’s test was used for correlations and linear regression for representation. *p*-value < 0.05 was considered significant and represented by the following symbols: * *p* < 0.05; ** *p* < 0.01; *** *p* < 0.001; and **** *p* < 0.0001.

## Data Availability

The raw data supporting the conclusions of this article will be made available by the authors on request.

## Supplementary Materials

The following supporting information can be downloaded at https://www.mdpi.com/article/10.3390/ijms25168569/s1.
