# Supplementary material for: Heterogeneous IL-9 Production by Circulating Skin-Tropic and Extracutaneous Memory T Cells in Atopic Dermatitis Patients
Source: Int J Mol Sci. 2024 Aug 6;25(16):8569. doi: 10.3390/ijms25168569 (PMC11354683; doi:10.3390/ijms25168569)
Supplement: Supplementary file 1 [file ijms-25-08569-s001.zip › ijms-3122849-supplementary.pdf]

SUPPORTING INFORMATION

Figure S1. Correlations between HDM-specific IgE levels and HDM-mediated production of Th2 cytokines. IL-4, IL-5, IL-13 and IL-31 (n=51) levels (pg/mL) produced by CLA^+/Epi and CLA^-/Epi cocultures after 5 days of activation with HDM were correlated with HDM-specific IgE plasma levels. CLA, cutaneous lymphocyte-associated antigen; Epi, epidermal cells; HDM, house dust mite; OD, optical density. ns: not significant ; **p <.01; ***p <.001.

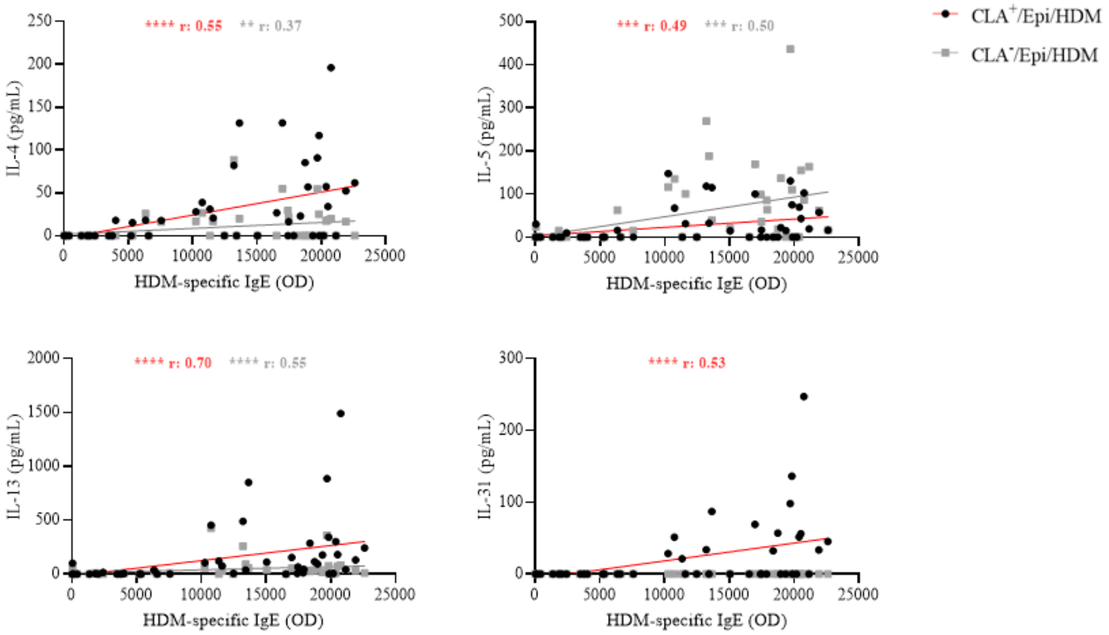

Figure S2. In response to HDM, CLA$^+$ and CLA$^-$ memory T cells produce IL-9 in a time-dependent manner. IL-9 cytokine levels (pg/mL) were measured in cocultures of CLA$^+$ and CLA- T cells after one, two, four and five days in basal conditions (M) or stimulated with HDM. Data from three representative experiments. CLA, cutaneous lymphocyte-associated antigen; Epi, epidermal cells; HDM, house dust mite.

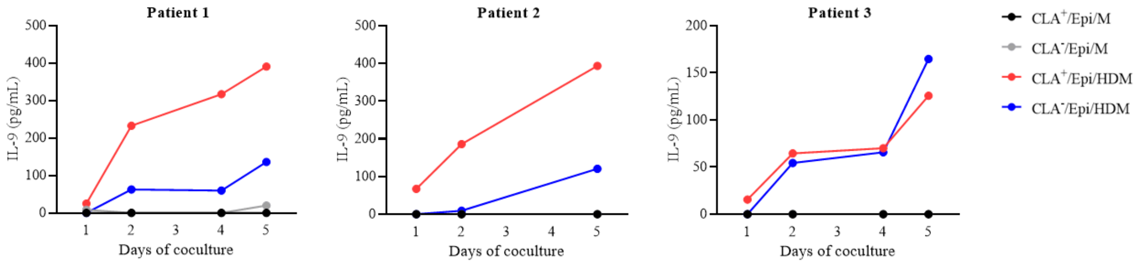

Figure S3. HDM- and SEB-induced IL-9 responses by circulating memory CLA$^+$ and CLA$^-$ T cells are strongly associated in AD patients. Correlation of IL-9 production by HDM-activated CLA$^{+/-}$/ T-cell cocultures with IL-9 production by SEB-activated CLA$^{+/-}$/ T-cell cocultures from AD patients (n=41). AD, atopic dermatitis; CLA, cutaneous lymphocyte-associated antigen; Epi, epidermal cells; HDM, house dust mite; SEB, staphylococcal enterotoxin B. **p <.01; ****p <.0001.

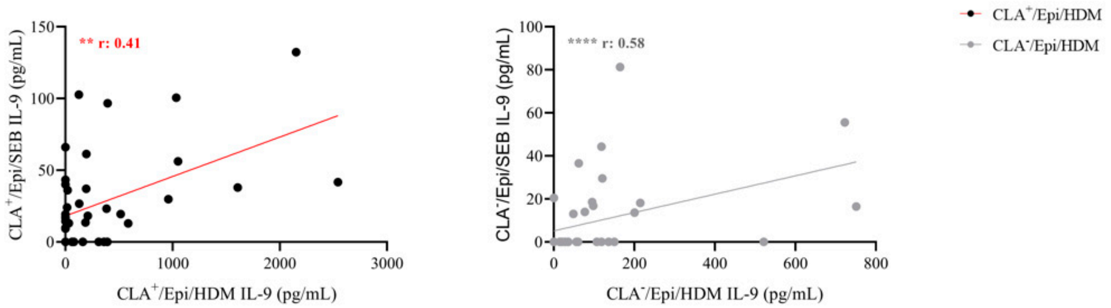

Table S1. IL-9 measured in 5-day supernatants from CLA^+ and CLA^- T-cell AD-derived cocultures activated with HDM were correlated with clinical characteristics. CLA, cutaneous lymphocyte-associated antigen; EASI, eczema area and severity index; Epi, epidermal cells; HDM, house dust mite; IGA, investigator global assessment; SCORAD, scoring atopic dermatitis; VAS, visual analogue scale.

	**CLA^+/Epi/HDM IL-9 (pg/mL)**			**CLA^-/Epi/HDM IL-9 (pg/mL)**		
**Clinical characteristics**	**R Spearman**	**ρ-value**	**N**	**R Spearman**	**ρ-value**	**N**
SCORAD	0.02	.93	41	-0.11	.51	41
EASI	0.16	.26	50	0.03	.81	50
IGA	0.23	.11	50	-0.01	.96	50
Pruritus (VAS)	0.04	.77	51	0.11	.46	51
Eosinophilia $(x10)^3/\mu l$	0.18	.24	45	-0.05	.73	45
Age	-0.13	.36	51	-0.25	.07	51
Years since diagnosis	-0.08	.56	50	-0.10	.51	50
Age at disease onset	0.07	.65	50	-0.03	.85	50

Table S2. IL-9 measured in 5-day supernatants from CLA^{+} and CLA^{-} T-cell AD-derived cocultures activated with HDM were correlated with clinical characteristics in patients that produce IL-9. CLA, cutaneous lymphocyte-associated antigen; EASI, eczema area and severity index; Epi, epidermal cells; HDM, house dust mite; IGA, investigator global assessment; OD, optical density; SCORAD, scoring atopic dermatitis; VAS, visual analogue scale.

	CLA^{+} producers IL-9 (pg/mL)			CLA^{-} producers IL-9 (pg/mL)		
**Clinical characteristics**	**R Spearman**	**ρ-value**	**N**	**R Spearman**	**ρ-value**	**N**
SCORAD	-0.26	.23	23	-0.02	.92	20
EASI	-0.09	.64	29	0.02	.94	26
IGA	0.15	.45	29	-0.27	.19	26
Pruritus (VAS)	-0.03	.87	30	-0.07	.75	27
Eosinophilia $(x10)^{3}/\mu l$	0.06	.79	25	-0.38	.08	22
Age	-0.13	.49	30	-0.14	.48	27
Age at disease onset	0.21	.27	30	0.22	.29	26
HDM-sp IgE (OD)	0.33	.08	29	-0.06	.75	26
Total IgE (kU/L)	0.21	.28	29	-0.13	.52	26

Table S3. Differences in cytokine and chemokine production and clinical characteristics between IL-9 producers and no producers AD patients described the CLA$^+$ T-cell response to HDM. Continuous variables are presented as medians (25th-75th percentiles) and categorical variables are presented as counts (percentages). Bold values indicate significant data. CLA, cutaneous lymphocyte-associated antigen; EASI, eczema area and severity index; HDM, house dust mite, IGA, investigator global assessment; LDH, lactate dehydrogenase; OD, optical density; SEB, staphylococcal enterotoxin B; SCORAD, scoring atopic dermatitis; VAS, visual analogue scale.

		CLA$^+$ producers		CLA$^+$ non-producers		
		**Median (25 – 75% percentiles) or Total number (%)**	**N**	**Median (25 – 75% percentiles) or Total number (%)**	**N**	**ρ-value**
Cytokines (pg/mL)	IL-13	113.60 (41.61-289.20)	30	0.00 (0.00-4.49)	22	**<.0001**
	IL-4	29.60 (0.00-66.86)	30	0.00 (0.00-0.00)	22	**<.0001**
	IL-5	26.27 (0.00-71.08)	30	0.00 (0.00-0.00)	22	**<.0001**
	IL-31	24.98 (0.00-52.74)	30	0.00 (0.00-0.00)	22	**<.0001**
	IL-17A	9.76 (0.00-46.01)	30	0.00 (0.00-0.00)	22	**<.0001**
	IL-22	36.44 (0.00-136.70)	26	0.00 (0.00-0.00)	22	**<.0001**
	IL-21	0.00 (0.00-19.34)	27	0.00 (0.00-0.00)	19	**.03**
	IFN-g	0.00 (0.00-28.80)	30	0.00 (0.00-0.00)	19	**.001**
	CCL17	128.70 (1.11-277.30)	11	27.59 (11.83-48.60)	11	.42
	CCL22	1514 (119.90-6408)	11	655.3 (307.9-1009)	11	.12
Clinical characteristics	Age	35.50 (25.50-46.00)	30	36.00 (27.00-46.50)	21	.65
	Years since diagnosis	24.00 (13.00-32.00)	30	21.00 (5.38-29.50)	20	.44
	SCORAD	60.00 (50.00-64.20)	23	54.50 (40.15-68.93)	18	.52
	EASI	26.60 (19.75-33.00)	29	18.00 (11.80-31.60)	21	.15
	IGA	4.00 (3.00-4.00)	29	3.00 (2.50-4.00)	21	.17
	Pruritus (VAS)	7.00 (6.75-9.00)	30	7.00 (6.00-8.25)	21	.63
	Eosinophilia $(x10)^3/\mu l$	0.60 (0.28-1.09)	25	0.50 (0.25-0.62)	20	.24
	HDM-sp IgE (OD)	17874 (11484-20006)	29	3632 (131.30-6412)	22	**<.0001**
	S. aureus-sp IgE (OD)	0.24 (0.00-0.56)	26	0.15 (0.00-0.82)	19	.77
	SEB-sp IgE (OD)	0.00 (0.00-0.17)	26	0.00 (0.00-0.00)	19	.41
	Total IgE (kU/L)	2000 (426 - 4776)	29	399,70 (94,05 - 1307)	22	**.002**
	Serum LDH (U/L)	225.00 (153.00-270.00)	15	150.00 (108.00-206.00)	15	.05
	Gender (male, n)	18 (62.07)	29	5 (23.81)	21	**.01**
	Atopic comorbidities, n	23 (82.14)	28	14 (66.67)	21	.32
	Allergic rhinitis, n	18 (64.29)	28	11 (52.38)	21	.56
	Allergic asthma, n	11 (39.29)	28	9 (42.86)	21	>.99
	Allergic conjunctivitis, n	14 (50.00)	28	7 (33.33)	21	.33
	Food allergy, n	7 (25.00)	28	5 (26.32)	19	>.99

Table S4. Differences in cytokine and chemokine production and clinical characteristics between IL-9 producers and no producers AD patients described the CLA$^-$ T-cell response to HDM. Continuous variables are presented as medians (25th-75th percentiles) and categorical variables are presented as counts (percentages). Bold values indicate significant data. CLA, cutaneous lymphocyte-associated antigen; EASI, eczema area and severity index; HDM, house dust mite, IGA, investigator global assessment; LDH, lactate dehydrogenase; OD, optical density; SEB, staphylococcal enterotoxin B; SCORAD, scoring atopic dermatitis; VAS, visual analogue scale.

		CLA$^-$ producers		CLA$^-$ non-producers		
		**Median (25 – 75% percentiles) or Total number (%)**	**N**	**Median (25 – 75% percentiles) or Total number (%)**	**N**	**ρ-value**
Cytokines (pg/mL)	IL-13	48.70 (27.22-71.75)	27	0.00 (0.00-0.00)	25	**<.0001**
	IL-4	16.63 (0.00-25.22)	27	0.00 (0.00-0.00)	25	**<.0001**
	IL-5	85.81 (24.05-137.20)	27	0.00 (0.00-0.00)	25	**<.0001**
	IL-31	0.00 (0.00-0.00)	27	0.00 (0.00-0.00)	25	**>.99**
	IL-17A	0.00 (0.00-7,13)	27	0.00 (0.00-0.00)	25	**.0001**
	IL-22	0.00 (0.00-40.21)	24	0.00 (0.00-0.00)	21	**.01**
	IL-21	0.00 (0.00-0.00)	25	0.00 (0.00-0.00)	21	.11
	IFN-g	0.00 (0.00-124.60)	27	0.00 (0.00-0.00)	25	**.002**
	CCL17	68.13 (4.60-239.80)	12	19.46 (1.29-38.78)	10	.18
	CCL22	1365 (164.60-2810)	12	444.40 (28.11-490.60)	10	.07
Clinical characteristics	Age	34.0 (26.00-44.00)	27	41.00 (29.25-49.50)	24	.19
	Years since diagnosis	23.50 (12.50-30.50)	26	22.50 (8.00-32.25)	24	>.99
	SCORAD	59.45 (50.00-61.87)	20	60.00 (41.15-75.85)	21	.49
	EASI	25.00 (18.33-32.00)	26	22.75 (13.00-36.28)	24	.81
	IGA	3.00 (3.00-4.00)	26	3.00 (3.00-4.00)	24	.62
	Pruritus (VAS)	7.00 (7.00-9.00)	27	7.50 (6.00-8.00)	24	.31
	Eosinophilia $(x10)^3/\mu l$	0.57 (0.30-0.90)	22	0.53 (0.24-0.93)	23	.71
	HDM-sp IgE (OD)	17668 (12811-19733)	26	3497 (260.50-8974)	25	**<.0001**
	S. aureus-sp IgE (OD)	0.26 (0.00-0.63)	24	0.15 (0.00-0.68)	21	.90
	SEB-sp IgE (OD)	0.00 (0.0-0.10)	24	0.00 (0.00-0.12)	21	.79
	Total IgE (kU/L)	1575 (334.90-3923)	26	568 (107-1463)	25	**.002**
	Serum LDH (U/L)	219.50 (140.30-263.30)	16	175.50 (112.50-210.30)	14	.19
	Gender (male, n)	14 (53.85)	26	9 (37.50)	24	.27
	Atopic comorbidities, n	18 (69.23)	26	18 (75.00)	24	.76
	Allergic rhinitis, n	14 (56.00)	24	15 (62.50)	24	.77
	Allergic asthma, n	9 (36.00)	24	11 (45.89)	24	.57
	Allergic conjunctivitis, n	11 (44.00)	24	10 (41.67)	24	>.99
	Food allergy, n	5 (20.00)	25	7 (31.82)	22	.50

Table S5. IL-9 measured in 24h supernatants from CLA$^+$ and CLA$^-$ T-cell AD-derived cocultures activated with SEB were correlated with clinical characteristics. Bold values indicate significant data. CLA, cutaneous lymphocyte-associated antigen; EASI, eczema area and severity index; Epi, epidermal cells; IGA, investigator global assessment; OD, optical density; SCORAD, scoring atopic dermatitis; SEB, staphylococcal enterotoxin B; sp, specific; VAS, visual analogue scale.

	**CLA$^+$/Epi/SEB IL-9 (pg/mL)**			**CLA$^-$/Epi/SEB IL-9 (pg/mL)**		
**Clinical characteristics**	**R Spearman**	**ρ-value**	**N**	**R Spearman**	**ρ-value**	**N**
SCORAD	-0.25	.14	35	-0.12	.48	35
EASI	0.05	.72	49	0.18	.23	49
IGA	0.03	.81	48	-0.01	.97	48
Pruritus (VAS)	0.06	.68	48	0.02	.88	48
Eosinophilia $(x10)^3/\mu l$	-0.21	.18	42	-0.19	.23	42
Age	-0.13	.36	49	-0.30	**.03**	49
Years since diagnosis	-0.09	.57	46	-0.19	.21	46
Age at disease onset	0.03	.86	49	-0.04	.79	49
S. aureus-sp IgE (OD)	0.00	.99	48	-0.05	.72	48
SEB-sp IgE (OD)	0.01	.95	48	-0.15	.31	48

Table S6. IL-9 measured in 24h supernatants from CLA$^+$ and CLA$^-$ T-cell AD-derived cocultures activated with SEB were correlated with clinical characteristics in patients that produce IL-9. CLA, cutaneous lymphocyte-associated antigen; EASI, eczema area and severity index; Epi, epidermal cells; IGA, investigator global assessment; OD, optical density; SCORAD, scoring atopic dermatitis; SEB, staphylococcal enterotoxin B; VAS, visual analogue scale.

	CLA$^+$ producers IL-9 (pg/mL)			CLA$^-$ producers IL-9 (pg/mL)		
**Clinical characteristics**	**R Spearman**	**ρ-value**	**N**	**R Spearman**	**ρ-value**	**N**
SCORAD	-0.31	.18	21	-0.07	.88	9
EASI	0.04	.83	34	0.36	.16	17
IGA	-0.06	.45	33	-0.16	.55	16
Pruritus (VAS)	-0.10	.57	33	-0.17	.50	17
Eosinophilia $(x10)^3/\mu l$	-0.09	.65	28	-0.42	.16	13
Age	-0.02	.93	34	0.31	.22	17
Age at disease onset	0.24	.19	33	0.30	.26	16
S. aureus-sp IgE (OD)	-0.10	.59	34	-0.03	.92	17
SEB-sp IgE (OD)	-0.13	.48	34	-0.35	.17	17
Total IgE (kU/L)	0.06	.73	33	-0.36	.16	17

Table S7. Differences in cytokine and chemokine production and clinical characteristics between IL-9 producers and no producers AD patients described the CLA$^+$ T-cell response to SEB. Continuous variables are presented as medians (25th-75th percentiles) and categorical variables are presented as counts (percentages). Bold values indicate significant data. CLA, cutaneous lymphocyte-associated antigen; EASI, eczema area and severity index; HDM, house dust mite, IGA, investigator global assessment; LDH, lactate dehydrogenase; OD, optical density; SEB, staphylococcal enterotoxin B; SCORAD, scoring atopic dermatitis; VAS, visual analogue scale.

		**CLA$^+$ producers**		**CLA$^+$ non-producers**		
		**Median (25 – 75% percentiles) or Total number (%)**	**N**	**Median (25 – 75% percentiles) or Total number (%)**	**N**	**ρ-value**
Cytokines (pg/mL)	IL-13	73.41 (41.32-102.40)	35	32.57 (19.51-63.19)	15	**.005**
	IL-4	48.96 (28.08-92.67)	35	29.17 (14.13-36.42)	15	**.003**
	IL-5	0.00 (0.00-12.78)	35	0.00 (0.00-0.00)	15	**.03**
	IL-31	0.00 (0.00-6.28)	35	0.00 (0.00-0.00)	15	**.09**
	IL-17A	114.10 (61.89-182.60)	35	58.18 (24.05-104.80)	15	**.01**
	IL-22	146.40 (57.81-247.90)	34	33.80 (0.00-79.29)	15	**.003**
	IL-21	28.35 (17.53-38.93)	35	16.54 (0.00-26,95)	15	**.01**
	IFN-g	366.20 (213.20-925.30)	35	214.20 (114.40-644.50)	15	**.11**
Clinical characteristics	Age	34.00 (25.75-45.25)	34	43.00 (27.00-50.00)	15	.39
	Years since diagnosis	22.00 (15.00-32.00)	33	20.00 (5.00-32.00)	15	.36
	SCORAD	57.34 (37.50-62.66)	21	59.82 (49.31-71.25)	14	.32
	EASI	25.00 (16.75-31.40)	34	22.00 (17.80-32.00)	15	.83
	IGA	3.50 (3.00-4.00)	33	3.00 (3.00-4.00)	15	.61
	Pruritus (VAS)	8.00 (7.00-9.00)	33	7.00 (6.00-9.00)	15	.38
	Eosinophilia $(x10)^3/\mu l$	0.46 (0.15-0.71)	28	0.54 (0.30-1.03)	14	.19
	S. aureus-sp IgE (OD)	0.25 (0.02-0.60)	34	0.13 (0.00-0.84)	14	.66
	SEB-sp IgE (OD)	0.00 (0.00-0.23)	34	0.00 (0.00-0.05)	14	.56
	HDM-sp IgE (OD)	13213 (3497-17911)	35	8553 (47.75-19487)	14	.49
	Total IgE (kU/L)	844.90 (331.40-2000)	35	401.20 (77.48-3889)	14	.50
	Serum LDH (U/L)	205.00 (146.80-237.00)	26	197.00 (103.00-252.00)	7	.60
	Gender (male, n)	16 (50.50)	32	7 (50.00)	14	>.99
	Atopic comorbidities, n	23 (69.70)	33	10 (66.67)	15	>.99
	Allergic rhinitis, n	17 (51.52)	33	8 (53.33)	15	>.99
	Allergic asthma, n	13 (39.39)	33	5 (33.33)	15	.76
	Allergic conjunctivitis, n	12 (36.36)	33	8 (53.33)	15	.35
	Food allergy, n	5 (16.13)	31	4 (28.57)	14	.43

Table S8. Differences in cytokine and chemokine production and clinical characteristics between IL-9 producers and no producers AD patients described the CLA^- T-cell response to SEB. Continuous variables are presented as medians (25th-75th percentiles) and categorical variables are presented as counts (percentages). Bold values indicate significant data. CLA, cutaneous lymphocyte-associated antigen; EASI, eczema area and severity index; HDM, house dust mite, IGA, investigator global assessment; LDH, lactate dehydrogenase; OD, optical density; SEB, staphylococcal enterotoxin B; SCORAD, scoring atopic dermatitis; VAS, visual analogue scale.

		**CLA^- producers**		**CLA^- non-producers**		
		**Median (25 – 75% percentiles) or Total number (%)**	**N**	**Median (25 – 75% percentiles) or Total number (%)**	**N**	**ρ-value**
Cytokines (pg/mL)	IL-13	31.45 (16.95-69.33)	17	7.68 (0.00-13.83)	33	**<.0001**
	IL-4	36.33 (18.86-36.33)	17	0.00 (0.00-17.70)	33	**<.0001**
	IL-5	31.80 (10.42-44.55)	17	0.00 (0.00-0.00)	33	**<.0001**
	IL-31	0.00 (0.00-0.00)	17	0.00 (0.00-0.00)	33	**>.99**
	IL-17A	43.65 (21.95-58.16)	17	8.52 (0.00-23.29)	33	**<.0001**
	IL-22	91.68 (54.68-144.60)	17	0.00 (0.00-26.58)	32	**<.0001**
	IL-21	26.15 (8.72-32.73)	17	0.00 (0.00-9.36)	33	**<.0001**
	IFN-g	607.30 (431.40- 1463)	17	365.90 (110.00-678.00)	33	**.003**
Clinical characteristics	Age	27.00 (24.00-37.50)	17	40.00 (28.25-50.00)	32	**.04**
	Years since diagnosis	20.00 (13.50-24.75)	16	23.50 (11.25-35.00)	32	.31
	SCORAD	57.34 (32.50-61.16)	9	59.82 (45.95-68.08)	26	.50
	EASI	27.70 (20.50-32.00)	17	22.10 (16.25-28.58)	32	.32
	IGA	3.00 (3.00-4.00)	16	3.25 (3.00-4.00)	32	.95
	Pruritus (VAS)	8.00 (7.00-9.00)	17	7.00 (6.00-9.00)	31	.76
	Eosinophilia $(x10)^3$/µl	0.32 (0.00-0.80)	13	0.00 (0.00-0.84)	29	.40
	S. aureus-sp IgE (OD)	0.23 (0.00-0.60)	17	0.25 (0.00-0.77)	31	.73
	SEB-sp IgE (OD)	0.00 (0.00-0.08)	17	0.00 (0.00-0.23)	31	.48
	HDM-sp IgE (OD)	16506 (3784-18329)	17	6470 (2244-18800)	32	.24
	Total IgE (kU/L)	1326 (542.50-2616)	17	417.50 (118.30-2245)	32	.14
	Serum LDH (U/L)	223.00 (153.00-270.00)	15	179.50 (112.50-234.00)	18	.08
	Gender (male, n)	9 (56.25)	16	15 (48.39)	31	.76
	Atopic comorbidities, n	11 (68,75)	16	22 (66,67)	32	>.99
	Allergic rhinitis, n	8 (50,00)	16	17 (53,13)	32	>.99
	Allergic asthma, n	5 (31,25)	16	13 (40,63)	32	.75
	Allergic conjunctivitis, n	7 (43,75)	16	13 (40,63)	32	>.99
	Food allergy, n	2 (14,29)	14	7 (22,58)	31	.70

Table S9. Patients' clinical data. Categorical variables are presented as counts (percentages) and numerical variables are presented as medians (25th-75th percentiles). AD, atopic dermatitis; EASI, eczema area and severity index; HDM, house dust mite; IGA, investigator's global assessment; LDH, lactate dehydrogenase; OD, optical density; SCORAD, scoring atopic dermatitis; SEB, staphylococcal enterotoxin B; sp, specific; VAS, visual analogue scale.

**Clinical characteristics**	**Patients with AD**	**N**
Age	35.00 (26.00-45.50)	61
Male gender, n (%)	27 (45.76%)	59
Years since AD diagnosis	22.50 (13.00-31.50)	60
Age at disease onset	7.50 (1.25-27.00)	60
SCORAD	59.27 (40.20-65.00)	47
EASI	23.30 (16.00-32.00)	59
IGA	3.00 (3.00-4.00)	60
Pruritus (VAS)	7.50 (6.25-9.00)	60
Eosinophilia $(x10)^3/\mu l$	0.51 (0.24-0.78)	54
HDM-sp IgE (OD)	10273 (1930-17893)	61
Total IgE (kU/L)	780.80 (205.40-2752.00)	61
S. aureus-sp IgE (OD)	0.24 (0.00-0.64)	52
SEB-sp IgE (OD)	0.00 (0.00-0.15)	52
Serum LDH (U/L)	200.50 (130.30-235.50)	36
Atopic comorbidities, n (%)	44 (72.13%)	61
Allergic rhinitis, n (%)	33 (55.93%)	59
Allergic asthma, n (%)	23 (38.98%)	59
Allergic conjunctivitis, n (%)	24 (40.68%)	59
Food allergy, n (%)	15 (26.32%)	57

Table S10. Control subjects' clinical data. Categorical variables are presented as counts (percentages) and numerical variables are presented as medians (25th-75th percentiles).

**Clinical characteristics**	**Control subjects**	**N**
Age	54.00 (35.00-76.00)	25
Male gender, n (%)	15 (60.00%)	25
